# A Rare Case of Hypopharyngeal Screw Migration after Spine Stabilization with Plating

**DOI:** 10.1155/2013/475285

**Published:** 2013-06-09

**Authors:** G. Salis, B. Pittore, G. Balata, C. Bozzo

**Affiliations:** ^1^Otorhinolaryngology Department, “P. Dettori Hospital,” Tempio Pausania, Italy; ^2^Otorhinolaryngology Department, “S. Francesco Hospital,” via Mannironi 1, 08100 Nuoro, Italy

## Abstract

Anterior cervical spine fusion and stabilization with plating are well-established surgical procedures for the treatment of myelopathy, cervical spine traumas, and spinal infectious diseases. Various complications have been described in the literature, more frequently, intraoperative bleeding, peri- or postoperative hypopharyngeal, and/or esophageal ruptures with mediastinal deep infection and loosening and extrusion of the screws from the plating. Screw migration has also been observed as a complication of the procedure, either early in the postoperative period or delayed, even after many years. In some instances, the esophageal perforation can recover spontaneously with absence of complications, even if a case of plate failure and graft migration with lethal sudden airways obstruction has been reported. We describe a case of hypopharyngeal screw migration after cervical spine stabilization with plating never described before in the literature.

## 1. Introduction

Due to the improved safety and outcomes, anterior surgical approaches to the cervical spine have gained popularity in the last decade. More commonly, the indications for this procedure are represented by cervical degenerative, neoplastic, and traumatic lesions.

Several complications are reported, mostly due to bleeding and rupture of hypopharyngeal and/or esophageal walls with mediastinal deep infection and loosening and extrusion of the screws from the plating. Though rare, migration of the screw after plating has been reported [1–12], the site of migration being more frequently the esophagus.

## 2. Case Report

A 65-years-old lady was referred to us from the Emergency Department, due to odynophagia, and sense of foreign body in her throat suddenly occurred 7 days before without any other symptom. Three years before, the patient underwent surgery with cervical spine stabilization with plating for C5-C6 disc herniation with a good outcome and absence of complications, apart from moderate dysphagia in the early postoperative period. 

**Figure 1:**
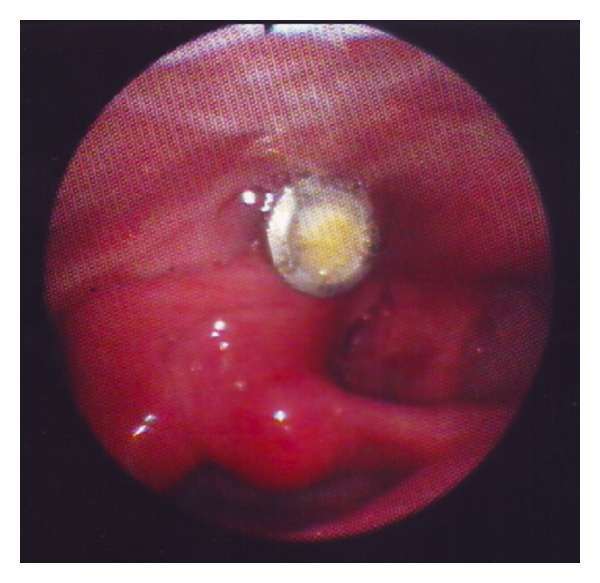
At laryngoscopy, the upper part of the screw is clearly visible in the postcricoid area, just above the entrance of left pyriform sinus.

**Figure 2:**
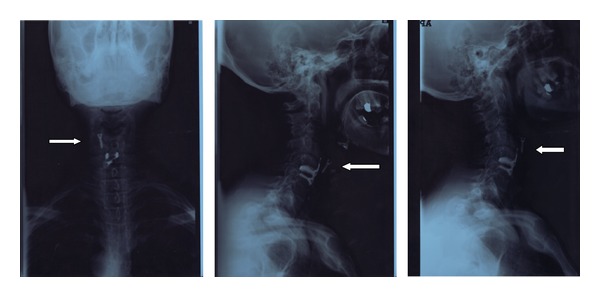
X-ray with contrast medium: the arrow clearly indicates the migration of the screw from the prosthesis in posteroanterior, right lateral, and left lateral projections.

A metallic foreign body (screw) was easily visible during laryngoscopy (Figure 1) in the postcricoid area, with only slight edema of the surrounding mucosa. An X-ray of the neck and a barium swallow confirmed the presence of the screw in the hypopharynx (Figure 2).

**Figure 3:**
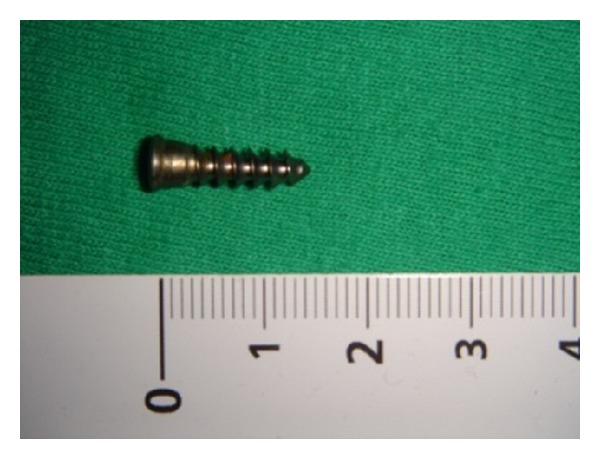
The screw after the removal, length 1.5 mm.

A microlaryngoscopy was then performed, the screw (Figure 3) being easily removed under general anesthesia by means of grasping forceps. At the end of the procedure, the hypopharynx and the proximal end of the esophagus were thoroughly assessed to rule out any perforation, and a nasogastric tube was positioned. The day after surgery a barium swallow was performed, showing the presence of a small fistula in the postcricoid area, with absence of symptoms or signs of infection. At day 6 the procedure was repeated with no evidence of fistula, so the patient was discharged and returned to a regular diet.

**Figure 4:**
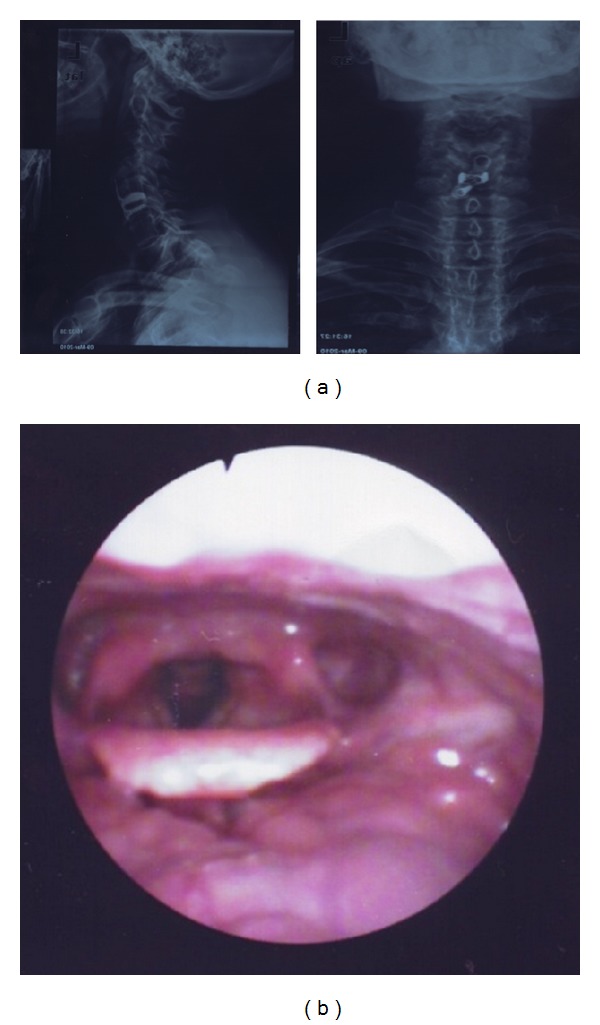
Radiographic and endoscopic controls after 3 years.

Radiographic (Figure 4(a)) and endoscopic (Figure 4(b)) controls 3 years after surgery were normal, with absence of symptoms. 

## 3. Discussion

In the last decade, there have been a growing development and refinement of surgical procedures indicated for spinal stabilization and fusion for cervical degenerative, neoplastic, and traumatic lesions. These procedures contemplate an anterior cervical access and the placement of plates and screws to guarantee an immediate stabilization and to improve the rate of bone fusion, avoiding the use of prolonged bracing with shorter hospital stay and early mobilization. 

Nonetheless, different complications can occur, such as cervical soft tissue swelling, hematomas, bleeding, breaches of the pharyngeal or esophageal walls, and, less often, screw displacement or migration. The latter, though rare, has been reported, the esophagus being the most frequent site of migration [1–12].

Esophageal injury after anterior cervical spine procedures is a rare complication. Sun described in a cohort of 2348 patients who underwent anterior cervical surgery only 5 esophageal perforations (0.2%) [13]. Screw migration can itself represent a cause of esophageal damage; Yee and Terry [3], Chataigner et al. [4], and Sahjpaul [9] reported few cases of esophageal perforations due to screw migration that occurred in the early postoperative period, while Pompili et al. [6] described a case of esophageal perforation 12 months after cervical spine procedure, and Fountas et al. [7] reported a case of screw extruded into the gastrointestinal tract 16 months after surgery.

Migration can also be observed further on. Gazzeri et al. [11] reported a case of delayed screw migration in the gastrointestinal tract 11 years after the procedure, while in our case, the interval between surgery and the onset of symptoms was shorter, though delayed as well (3 years).

In some instances, the esophageal perforation can spontaneously recover with absence of late complications. Yee and Terry [3] and Pompili et al. [6] recently described a case of screw migration through the esophagus and gastrointestinal tract with no significant morbidity, while Riew et al. [14] reported a case of plate failure and graft migration that caused fatal sudden airways obstruction.

Failure at the screw-bone interface occurs by either screw backout or screw cutout, the main predisposing factor that exposes to screw pullout appearing to be the initial malposition or suboptimal position of the screw.

Coe and Vaccaro [15], in their review of the literature, assessed the prevalence of screw and plate loosening between 0 and 15.4%, at various sites of the device: screw fracture between 0 and 13.3%, plate fracture between 0 and 6.7%, plate and graft displacement (with or without graft fracture) between 0 and 21.4%, and the prevalence of implant malposition (screws in discs, plating of unfused segments, etc.) between 0 and 12.5%.

As a matter of fact, in the most updated devices, the screws can be locked to avoid the risk of implant loosening and/or dislodgement with possible upper digestive tract damages.

## 4. Conclusion

Anterior cervical spine fusion and stabilization with plating are well-established surgical procedures for the treatment of degenerative, traumatic, or infective nature. Various complications have been described in the literature, either in the early postoperative period or delayed. Among the latter, hypopharyngeal and/or esophageal ruptures with mediastinal deep infection and loosening and extrusion of the screws from the plating have been reported. 

In case of screw migration, dysphagia can often represent the only symptom. Considering the long time required for the screw to migrate (up to several years after surgery), an accurate endoscopy and radiographic study of upper aerodigestive tract are in our opinion strongly advisable to promptly rule out a hypopharyngeal or esophageal foreign body when the patient's history reveals cervical spine previous surgery.
